# Promise and Uncertainty of Intermittent Fasting for Aging Populations

**DOI:** 10.1016/j.advnut.2026.100643

**Published:** 2026-05-01

**Authors:** Hassan S Dashti, Christine S Ritchie

**Affiliations:** 1 Department of Anesthesiology, Massachusetts General Hospital, Boston, MA, United States; 2 Division of Nutrition, Harvard Medical School, Boston, MA, United States; 3 Division of Sleep Medicine, Harvard Medical School, Boston, MA, United States; 4 Mongan Institute Center for Aging and Serious Illness, Massachusetts General Hospital, Boston, MA, United States; 5 Division of Palliative Care and Geriatric Medicine, Massachusetts General Hospital, Boston, MA, United States

Intermittent fasting (IF) is a dietary approach that alternates periods of energy restriction (or fasting) with periods of ad libitum food intake, typically structured across hours within a day (e.g., time-restricted eating) or days within a week (e.g., alternate-day fasting) [1]. IF has emerged as an effective strategy for weight management and metabolic health supported by large randomized trials, along with systematic reviews and meta-analyses, which show that IF produces weight loss and improvements in cardiometabolic health comparable to habitual diet and other dietary approaches [2]. As a result, time-restricted eating, alternate-day fasting, and other forms of IF have gained traction as alternatives to “traditional” continuous calorie restriction for their simplicity. Despite the growing popularity in the general population, the implications of IF in older adults remain less clearly defined, raising important concerns about safety.

Older adults represent a nutritionally vulnerable population [3]. Age-related changes, including reduced appetite, altered taste and smell, gastrointestinal changes, medication dependency, and multimorbidity, can predispose older adults to inadequate dietary intake and malnutrition [4]. Declines in muscle mass and strength, driven by anabolic resistance and alterations in protein metabolism, contribute to higher rates of sarcopenia, leading to frailty, disability, and loss of independence. Preservation of fat-free mass is therefore considered a key determinant of healthy aging, whereas unintentional weight loss may reflect loss of lean mass rather than adiposity. Any dietary approach that risks exacerbating muscle loss in older adults warrants scrutiny.

Recent epidemiological evidence has raised concerns about the association between shorter eating windows and longer fasting hours with increased mortality in older adults, questioning the role of IF in this population [5]. In the National Health and Nutrition Examination Survey, shorter eating windows (≤ 8 h/d) were found to be associated with higher risk of all-cause mortality showing a 30% or higher risk in older adults [6]. Similar findings were made in patient populations. In addition, several clinical nutrition practices emphasize small, frequent meals throughout the day for older adults to support nutritional adequacy [7]. These findings may appear to conflict with the reported benefits of IF and raise concerns about potential risks in its application in aging adults.

The scoping review by Santero et al. [8] provides a timely synthesis of the available evidence on IF in middle-aged and older adults (adults 40+ y) with overweight, obesity, and other metabolic diseases. Across 20 randomized controlled trials, the authors report that structured IF reduces body weight, fat mass, and waist circumference that are broadly comparable with continuous calorie restriction, and more consistent than habitual diet alone. Differences in fat-free mass between IF and the comparator diets were generally small, often <1 kg over short intervention periods (typically ≤12 wk), with no consistent signal that IF accelerates the loss of fat-free mass relative to continuous calorie restriction or standard care. Effects on insulin outcomes, protein intake, and combined IF with physical activity were limited and inconsistent, and functional performance and quality of life were rarely assessed, representing existing gaps. Overall, the findings suggest that, at least in the short term, IF may achieve anthropometric benefits without disproportionate compromise of lean mass.

As a scoping review, the work by Santero et al. is intended to map the existing evidence rather than provide definitive effect estimates. As noted by the authors, the substantial heterogeneity in study design, fasting protocols, comparator diets, and outcome measurements, limits the ability to draw firm conclusions. Furthermore, omissions and inconsistencies in key nutritional data substantially constrain the interpretation and real-world application of these research findings. Of the 20 trials, only 3 included adults aged ≥60 y, limiting the generalizability of findings to older populations, particularly those aged ≥70 y with multimorbidity and frailty. Based on the review, IF’s long-term effects in aging populations cannot be made at this time.

Findings from the review do not conflict with the epidemiological evidence on eating windows but warrant careful consideration of food timing in older adults. Although the clinical trials assessed structured, supervised IF, the epidemiological studies examined habitual eating windows, and results from one context cannot be directly extrapolated to the other. For some older adults, fasting protocols may inadvertently reinforce already limited eating patterns, increasing risk of inadequate energy and protein consumption. What may appear as beneficial weight loss could instead represent muscle mass loss and progress toward sarcopenia regardless of body size. These considerations underscore the importance of distinguishing intentional, deliberate, and structured dietary interventions, such as IF, from unintentional, illness- or aging-related anorexia or meal-skipping behaviors that frequently accompany aging due to physiological changes, multimorbidity, or medication burden. Therefore, careful monitoring of nutritional adequacy when applying IF in this population is key, and incorporating combined aerobic and resistance exercise during weight loss interventions is likely the most effective approach for improving functional status [3].

Taken together, the current evidence positions short-term IF as a potentially effective strategy for reducing excess weight and improving cardiometabolic health in aging populations. Its comparable efficacy to “traditional” continuous calorie restriction, combined with its relative simplicity, may offer practical advantages in real-world settings. However, the applicability of IF to individuals aged ≥70 y, those with sarcopenia, or those with multimorbidity remains largely unknown, as these populations are underrepresented in existing trials. Ultimately, the review by Santero et al. highlights the urgent need for future research that prioritizes longer-term interventions in older adults, rigorously assesses dietary intake and protein adequacy, and includes functional and patient-centered outcomes such as strength, mobility, and quality of life.

## Author contributions

Both authors wrote and approved the final article.

## Funding

The authors reported no funding received for this study.

## Conflict of interest

HSD is a consultant for Eli Lilly & Company and has received speaking fees from Option Care Health; these relationships are unrelated to the current work.
